# The definition and evaluation of uncoordinated involvement of multiple healthcare providers; “Polydoctoring” as a component of care fragmentation among patients which multimorbidity

**DOI:** 10.1002/jgf2.673

**Published:** 2024-01-13

**Authors:** Yuki Ohnishi, Satoshi Watanuki

**Affiliations:** ^1^ Sosa Municipal Hospital Sosa city Japan

To the editor,

We read with great interest the article by T Ando et al, and appreciate the authors' efforts to assess the influence of the uncoordinated involvement of multiple healthcare providers: “polydoctoring.” The analysis highlights that the involvement of multiple healthcare facilities in patient care is correlated with a higher likelihood of polypharmacy and increased outpatient medical costs.^1^ However, we would like to point out two concerns.

First, a significant issue in this study was that using the definition of “polydoctoring,” which refers only to having two or more regularly visited facilities, cannot appropriately evaluate the current situation in Japan. It might be unavoidable for today's elderly individuals in Japan to visit multiple medical institutions more than two. Historically, organ specialists played an important role in primary care settings in Japan, and we still have approximately 100,000 primary clinics run by organ specialists. The Japan Primary Care Association (JPCA) has started a training program to qualify doctors as General Practitioner/Family Physician specialists since 2017.^2^ Although JPCA expects these doctors to address a wider variety of common problems such as eye problems and osteoporosis, as well as common medical conditions,^3^ it is inevitable to face a transient lack of genuine primary care physicians who have completed a proper program. Therefore, in Japan, visiting multiple clinics is necessary for elderly people with coexisting chronic conditions that are beyond the scope of the primary care physicians they see. While the primary care system in Japan is still in development, thanks to universal access under the national health insurance system, elderly individuals can visit multiple medical facilities and enjoy health equity. There is no doubt that the establishment of the universal health insurance scheme in 1961 supports freedom to access medical facilities and services in Japan.^4^ It might be effective to consider the medical specialties visited when renewing the definition of polydoctoring.

In addition, the sample selection was problematic. The authors enrolled individuals only from an independent‐dwelling subset. Given the study result, if this survey were to include homebound elderly patients with multimorbidity, they would have fewer chances of receiving polypharmacy. Some elderly individuals, experiencing a decline in physical strength that makes it difficult to visit outpatient clinics, transition to home medical care, where their care should be consolidated. However, it was reported that the prevalence of inappropriate polypharmacy was 70% among older adults receiving home medical care.^5^ Inappropriate prescriptions do not always appear to be associated with care fragmentation.

Accordingly, we suggest that this study cannot accurately evaluate the impact of care fragmentation on patient outcomes because of the definition and sample selection. To understand the quality and efficiency of care for patients with multimorbidity, further research is required. We hope that by addressing the fragmentation of care, primary care in Japan will be improved more.

## CONFLICT OF INTEREST STATEMENT

The authors declare no conflict of interests for this article.

## ETHICS STATEMENT

The letter has been approved by the authors' institutional review board or equivalent committee.

## References

[1] Ando T , Sasaki T , Abe Y , Nishimoto Y , Hirata T , Haruta J , et al. Measurement of polydoctoring as a crucial component of fragmentation of care among patients with multimorbidity: cross‐sectional study in Japan. J Gen Fam Med. 2023;24(6):343–349. 38025930 10.1002/jgf2.651 PMC10646296

[2] Takamura A . The present circumstance of primary Care in Japan. Qual Prim Care. 2015;23(5):262–266.

[3] The Japan Primary Care Association JPCA . Family Medicine Expert Training Program. https://www.shin‐kateiiryo.primary‐care.or.jp/what‐system Accessed on December 4, 2023.

[4] Matsuda S . Health policy in Japan ‐ current situation and future challenges. JMA J. 2019;2(1):1–10. 33681508 10.31662/jmaj.2018-0016 PMC7930804

[5] Hamada S , Iwagami M , Sakata N , Hattori Y , Kidana K , Ishizaki T , et al. Changes in polypharmacy and potentially inappropriate medications in homebound older adults in Japan, 2015–2019: a Nationwide study. J Gen Intern Med. 2023;38:3517–3525. 10.1007/s11606-023-08364-4 37620717 PMC10713963

